# Video-Assisted Endoscopic Inguinal Lymph Node Dissection for Acral Melanoma

**DOI:** 10.7759/cureus.40136

**Published:** 2023-06-08

**Authors:** Jose A Rosales, Christian A Palacios, Tatiana Fernandez Trokhimtchouk, Luis F Flores, Álvaro Morillo Cox, Joseline K Crespo Martinez, Melissa German

**Affiliations:** 1 General Surgery, Universidad Internacional del Ecuador/Axxis Hospital, Quito, ECU; 2 Head and Neck Surgery, Hospital Carlos Andrade Marín, Quito, ECU; 3 Surgical Oncology, Hospital Carlos Andrade Marín, Quito, ECU

**Keywords:** amputation, surgical oncology, endoscopic, surgical procedure, lymphadenectomy, melanoma

## Abstract

This article discusses acral melanoma, a rare subtype of melanoma often presented at the later stages of the disease and is, thus, associated with poor survival rates, especially in patients with a lower socioeconomic status. Surgical resection is the primary treatment option for localized acral melanoma, while amputation is often necessary for tumors on the digits or the midfoot. Lymphadenectomy may be necessary for patients with regional lymph node involvement; however, the therapeutic role of dissection remains controversial. Here, we present the case of a 68-year-old man with acral melanoma who underwent a Lisfranc amputation and endoscopic groin lymph node dissection for ganglionic metastasis. In Ecuador, this is the first reported case of endoscopic groin lymphadenectomy for regional lymph node metastasis secondary to acral melanoma. The discussion explores the role of sentinel lymph node biopsy and the completion of lymph node dissection in managing regional lymph nodes in melanoma patients. This case study aims to contribute to the growing knowledge on acral melanoma, assess the need for better patient care, and analyze the role of minimally invasive techniques for inguinal lymph node dissections.

## Introduction

Melanoma is one of the most common malignancies worldwide, with a reported lifetime risk for females 1 in 44 and males 1 in 28. Moreover, it is the leading cause of mortality from cutaneous malignancy in the United States [1]. Acral melanoma is a rare subtype of melanoma and accounts for approximately 1-2% of all melanoma cases. It typically occurs on the palms of the hands, the feet' soles, and the digits' nail beds [2].

In 1977, Arrington et al. first reported a distinct subtype of skin melanocyte malignancy called acral lentiginous melanoma (ALM), characterized by its occurrence on the hands and feet. It is recommended that these lesions undergo a biopsy, which can be performed by a partial sampling of the mass; the most nodular portion provides the best Breslow depth, although there is a risk of inaccurate sampling, which can be overcome by performing a narrow margin excisional biopsy, yet this is not always practical in acral sites [3].

According to the depth of the tumor at the time of the biopsy, a wide local excision forms the basis of the ALM treatment. Clinical margins depend on the depth: for lesions in situ, the recommended margin is between 0.5 and 1 cm, for depths < 1 mm, the margin should be 1 cm, for depths between 1.01 and 2 mm, the clinical margin is 1 to 2 cm, and for a Breslow depth > 2 mm, a 2 cm clinical margin is required [2].

Acral melanoma is often diagnosed later than other forms, with up to 50% of cases presenting with regional or distant metastasis at the time of diagnosis. The 5-year survival rate for patients with acral melanoma varies depending on the stage at the time of the diagnosis, ranging from approximately 95% for diagnoses occurring in stage I of the disease to approximately 15% for those that occur at stage IV [4].

Surgical resection is the primary treatment option for localized acral melanoma, with amputation often necessary for tumors located on the digits or midfoot. However, lymphadenectomy may be necessary for patients with regional lymph node involvement. Indeed, multiple studies have evaluated the value of controlling regional nodal disease through surgical removal, and while prognostic significance exists for this treatment option, the survival benefits it offers are controversial [4]. 

In this report, we present the case of a 68-year-old man who presented acral melanoma in his right foot and underwent a Lisfranc amputation and endoscopic groin lymph node dissection for regional lymph node metastasis. The patient exhibited a lesion on the sole of his right foot, which had been initially misdiagnosed as a traumatic midfoot blister. However, the subsequent biopsy revealed it to be an acral melanoma, and there were palpable groin lymphadenopathies, which suggested regional lymph node involvement.

## Case presentation

**Figure 1:**
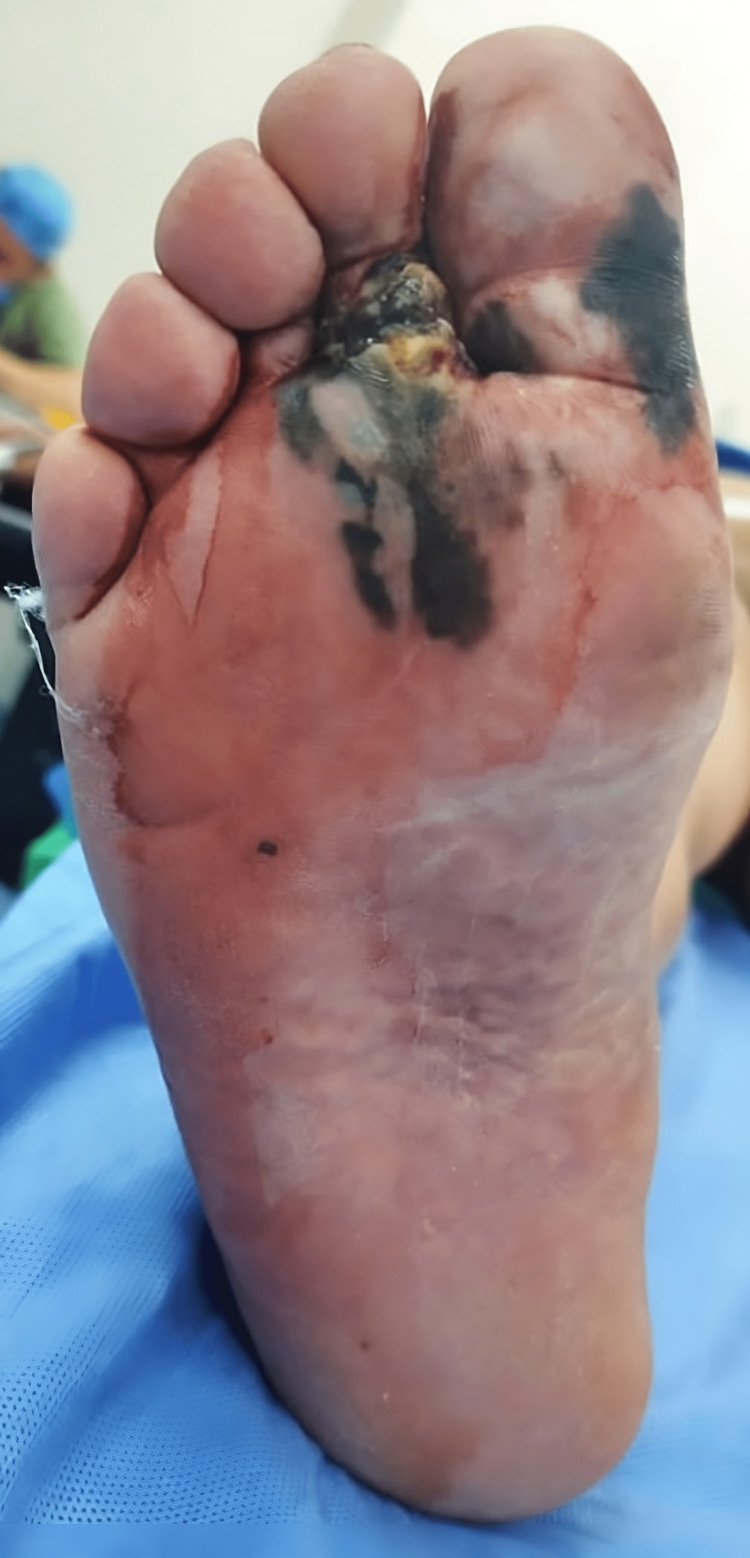
Clinical features of acral melanoma on the right foot

A 68-year-old male, who had a history of hypothyroidism and had undergone a laparoscopic cholecystectomy two years ago, presented with an 8-month history of a darkly pigmented lesion on his right foot. He had initially noted this lesion alongside a traumatic ulcer and blisters on the plantar aspect of the midfoot. A subsequent physical examination revealed a 3 cm exophytic, irregular mass on the sole, which involved the second toe, and a pigmented macule on the first toe (Figure 1). The lesion was described as painful and had become ulcerated while exhibiting color changes, episodes of bleeding, and reported growth. Upon exploration, palpable right inguinal lymphadenopathies with at least 3 nodes, each 1 cm in size, were also identified. An incisional biopsy of the mass revealed an invasive acral melanoma with a Breslow thickness of 4 mm, a dermal mitotic rate of 13/mm^2^, Clark level IV (invasion into the reticular dermis), and no lymphovascular or perineural invasion.

A full-body CT scan was performed, which showed no evidence of any distant metastasis; however, it did reveal the presence of enlarged nodes in the inguinal basin, and no iliac or obturator lymph node involvement was identified.

The patient was scheduled for a Lisfranc amputation and endoscopic inguinal lymph node dissection. A disarticulation at the tarsometatarsal level was performed using an arciform incision at the plantar and dorsal aspects of the right foot over the metatarsus and was completed without any complications. However, the lymphadenectomy discovered various darkened lymph nodes in the groin basin.

**Figure 2:**
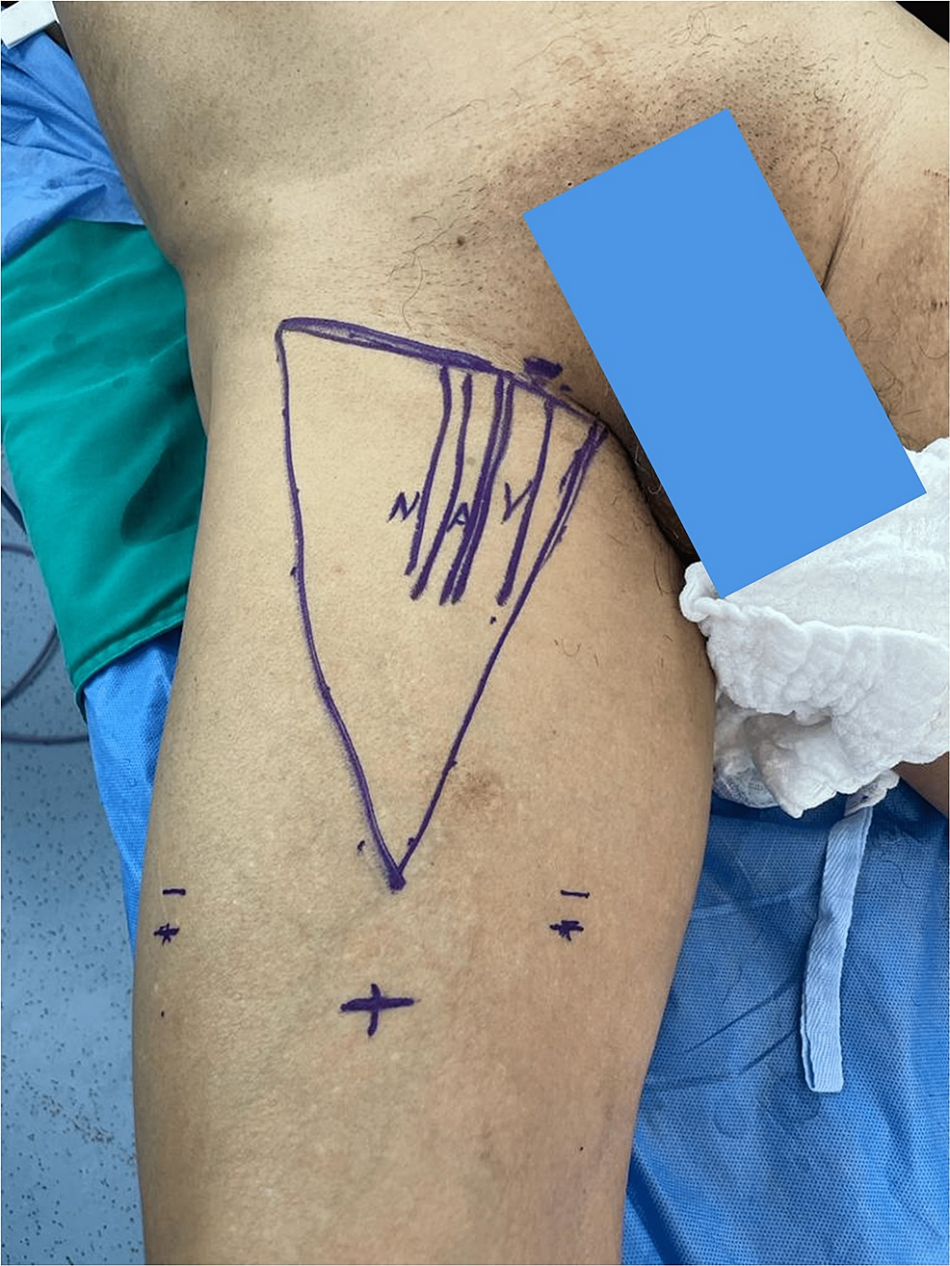
Demarcation of the femoral triangle. Trocar placement is shown.

The femoral triangle was demarcated, and a 10 mm incision was created, 6 cm distal from the triangle's apex, for the dissection of the prefascial/subcutaneous plane, trocar placement, and initial endoscopy. A total of 2 further 5 mm incisions in the medial and lateral aspects of the triangle were produced for the accessory trocars, which were placed under direct vision. Figure 2 shows the demarcation.

**Figure 3:**
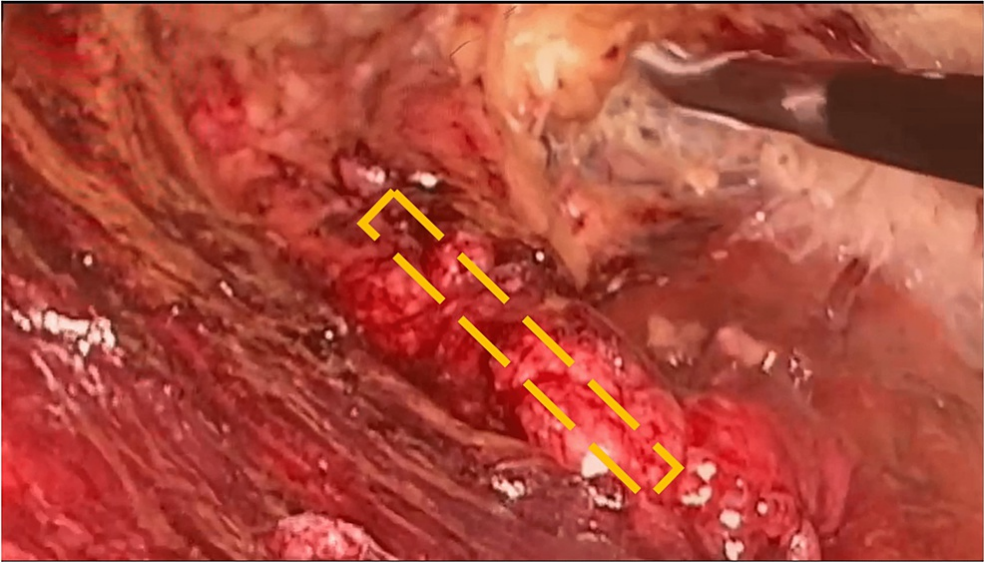
Endoscopic view of the dissection. The yellow-dashed line indicates the femoral artery.

The dissection started in the prefascial plane, from the medial and lateral muscles over to the sartorius and adductor longus muscles, and proceeded up until the identification of the femoral triangle, with the corresponding artery, vein, and nerve (Figure 3). Ganglionar dissection was conducted over these aforementioned structures, and the specimen was retrieved through a 3 cm incision below the inguinal ligament. Thereafter, meticulous hemostatic control was conducted, following which a closed-suction drain was placed, and the layers were sutured closed.

The pathology report confirmed the presence of a 4 x 4 cm invasive acral melanoma, with a mitotic index of 7 mm^2^, a Breslow thickness > 4 mm, of Clark level V (invasion of adipose tissue), no lymphovascular invasion, present perineural invasion, no satellitosis, free surgical margins, and no osseous compromise. Furthermore, it described six lymph nodes in the groin specimen that were all positive, while an extranodal extension was also present; however, a BRAF mutation was not detected. Thus, the diagnosis of stage III invasive acral melanoma with pT4b N3 M0 was concluded.

The patient underwent an uneventful postoperative recovery and was discharged on the fourth day after removing the drain, demonstrating adequate analgesic control and no signs of complications. Presently, the patient is awaiting adjuvant therapy.

## Discussion

In the current literature, three landmark randomized controlled trials guide lymph node dissection in melanoma: MSLT-I, DeCOG-SLT, and MSLT-II. These studies have discussed the management of regional lymph nodes and have demonstrated the use of sentinel lymph node (SLN) biopsy in providing paramount information for prognosis. Furthermore, they have addressed the therapeutic role of dissection [5].

MSLT-I confirmed that there was better regional disease control and longer disease-free survival in intermediate-thickness (Breslow depths of 1-4 mm) melanomas following early nodal treatment [6]. The German study, DeCOG-SLT, addressed the role of completion lymph node dissection (CLND) after a positive SLN, and although it was underpowered, it concluded that there was no benefit to survival outcomes from CLND and that it should not be recommended in cases of nodal micrometastases of 1 mm or smaller [7].

While there is no question regarding the prognostic role of performing an SLN biopsy, controversy remains over the therapeutic role of CLND. Indeed, the MSLT-II study evaluated whether CLND was necessary and reached the conclusion that observation alone alongside high-performance ultrasonography was safe; a finding that formed the basis for the National Comprehensive Cancer Network (NCCN) and American Society of Clinical Oncology (ASCO) recommendations on the management of metastatic melanoma regional nodal disease, in relation to the morbidity and complications associated with CLND [8,9].

Here, the patient presented palpable nodal disease, a common occurrence in people of low socioeconomic status treated in this public health tertiary center. According to local protocols, these patients are subject to lymph node dissection without an SLN biopsy.

The 2002 study by Hughes et al. presented data on the evaluation of various prognostic factors, including age, sex, tumor thickness, ulceration, lymph node involvement, and their number. The authors also discussed the surgical management of patients with palpable lymph node metastases, including dissection and the timing of surgery. The paper's main findings suggested that the patients with a lower tumor burden and fewer involved lymph nodes had a better prognosis than those with a higher tumor burden and more extensive ganglion involvement. The authors also found that early surgical intervention, such as immediate lymph node dissection, was associated with improved outcomes [10]. 

A study by Tobias-Machado et al. [11] described the video endoscopic inguinal lymphadenectomy (VEIL) technique, a minimally invasive resection of inguinal lymph nodes. This technique uses small incisions alongside endoscopy to identify the femoral triangle and the corresponding artery, vein, and nerve and to conduct a ganglion dissection over these structures. The authors reported favorable outcomes when using VEIL, including pain reduction, surgical time, and length of stay. Notably, this technique was performed on our case patient and, to the best of our knowledge, represents the first case reported in Ecuadorian literature. 

Another paper by Nabavizadeh [12] discussed inguinal lymph node dissection in the era of minimally invasive surgical techniques, including VEIL. The author described the advantages of minimally invasive techniques, including reduced morbidity and complications, decreased hospital stay, and quicker recovery periods. The author concluded that these techniques were safe and effective and should be considered in the appropriate clinical settings. Further, a series by Sommariva and Rossi [13] described the use of videoscopic inguinal lymphadenectomy as a novel approach for melanoma groin metastases. The authors reported on their experience using the technique, which they had performed on 20 patients with melanoma. They noted its favorable outcomes, including reduced hospital stays, less postoperative pain, and lower morbidity rates than traditional inguinal lymphadenectomy.

Overall, this case report underlines the importance of lymph node dissection in patients with melanoma and highlights the potential benefits of minimally invasive techniques, such as VEIL, in reducing morbidity and complications. However, it also raises questions regarding the appropriateness of local protocols that favor lymph node dissection without performing an SLN biopsy, particularly in low-income populations, and calls for further research to better understand the risks and benefits of different approaches to inguinal dissection in melanoma cases.

## Conclusions

Acral melanoma is a rare and often late-presenting subtype of melanoma. Surgical resection is the primary treatment of localized disease, with amputation in some instances. Whenever there is regional lymph node involvement, the performance of lymphadenectomy has a prognostic role and can potentially improve survival outcomes. The importance of controlling the regional nodal disease by surgical removal, however, is controversial, and there is still no consensus on the therapeutic role of CLND. Minimally invasive techniques are a safe option for nodal metastatic melanoma treatment, with oncologic results comparable to those of open inguinal lymph node dissection. The case presented in this report, where a patient with an acral melanoma who underwent a Lisfranc amputation and endoscopic groin lymph node dissection for the treatment of regional lymph node metastasis, is significant in Ecuadorian medical literature because it represents the first reported case of its kind. Our aim in documenting this case is to add to the expanding pool of information on acral melanomas and to improve the quality of medical treatment available for patients in Ecuador and worldwide.
